# Supplementary material for: ROCK2-induced metabolic rewiring in diabetic podocytopathy
Source: Commun Biol. 2022 Apr 8;5:341. doi: 10.1038/s42003-022-03300-4 (PMC8993857; doi:10.1038/s42003-022-03300-4)
Supplement: Supplementary file 2 — Supplementary Information [file 42003_2022_3300_MOESM2_ESM.pdf]

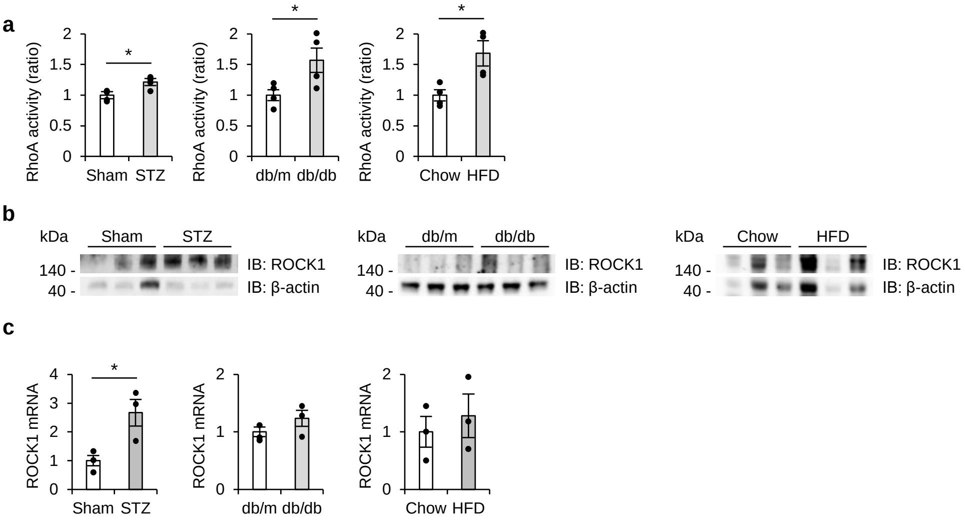

Supplementary Fig. 1: ROCK signaling pathway in animal models of diabetes. a, The RhoA activity in the renal cortex of STZ-injected mice, db/db mice, and mice treated with HFD (n = 4). **b**, ROCK1 levels in the renal cortex of animal models of diabetes (n = 3). **c**, The mRNA expression levels of ROCK1 in isolated glomeruli (n = 3). Data are represent the mean ± s.e.m.

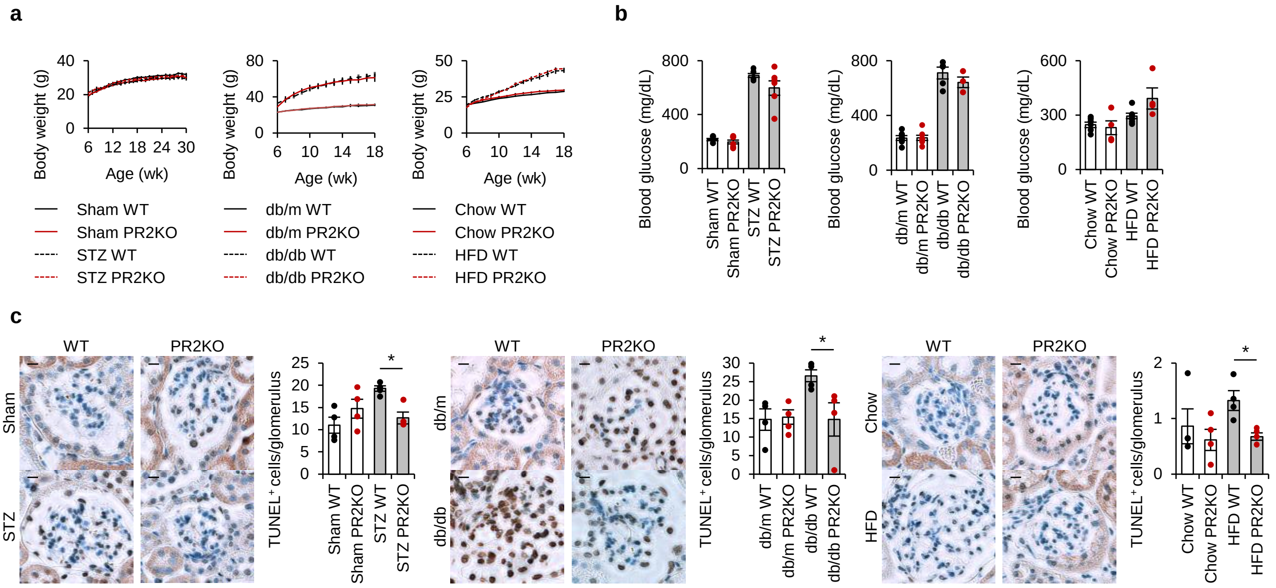

Supplementary Fig. 2: Characteristics of diabetic PR2KO mice. a, The body weight of STZ-injected, db/db, HFD-fed WT and PR2KO mice (*n* = 3-9). **b**, Blood glucose levels in three murine models of diabetes (*n* = 3-7). Data are represent the mean ± s.e.m. **c,** Cell death as detected by TUNEL assay in STZ-injected, db/db, HFD-fed WT and PR2KO mice. The scale bar on the top left represents 10 μm (*n* = 4). Nuclei were visualized using DAPI staining. Representative glomerular images and quantitative assessments of the number of cell death (number of TUNEL-positive cells per glomerulus) are demonstrated.

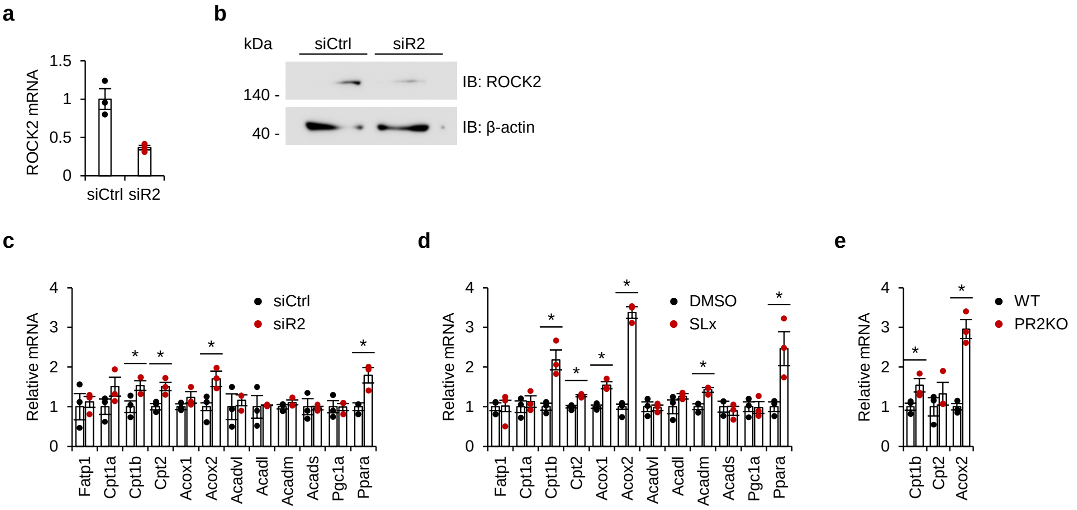

Supplementary Fig. 3: Characterization of ROCK2-null podocytes and effects of ROCK2 inhibition. The mRNA (**a**) and protein (**b**) expression levels of ROCK2 in podocytes treated with siRNA against ROCK2. Gene expression levels of fatty acid metabolism mediators in podocytes incubated with ROCK2 siRNA (**c**) and in podocytes treated with ROCK2 inhibitor SLx (**d**) (*n* = 3). **e**, Glomerular mRNA levels of Cpt1b, Cpt2, Acox2 in WT and PR2KO mice (*n* = 3). *p < 0.05. Data are represent the mean ± s.e.m.

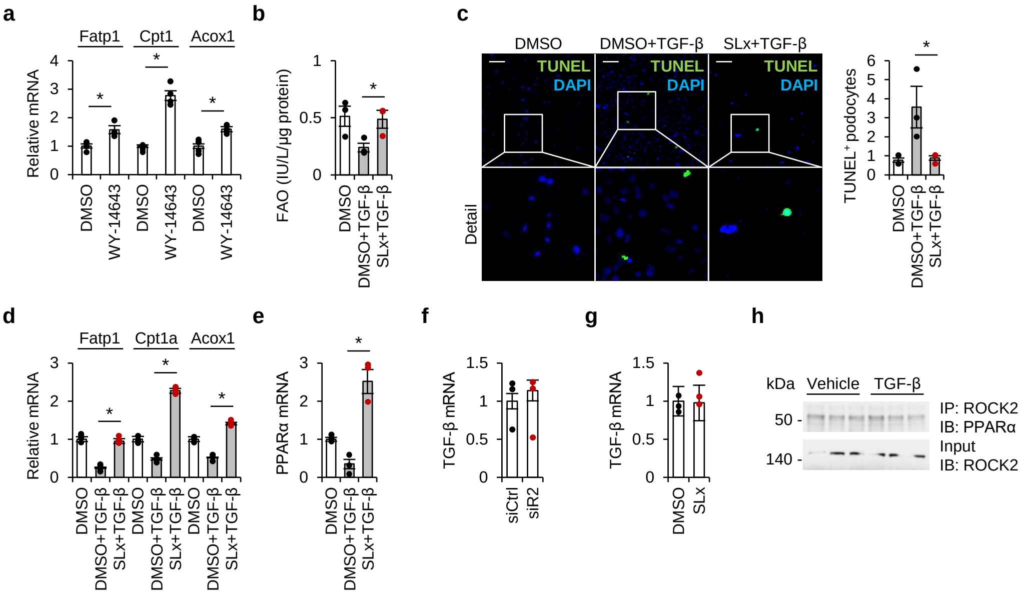

Supplementary Fig. 4: Pharmacological inhibition of ROCK2 improves fatty acid metabolism. a, The expression levels of fatty acid oxidation (FAO) regulators in podocytes treated with WY-14643, a PPARα agonist, or DMSO (*n* = 4). **b**, FAO was assessed using cell lysates obtained from podocytes treated with SLx-2119, a ROCK2 inhibitor, or DMSO (*n* = 3). **c**, Representative microphotographs and quantification of TUNEL-positive apoptotic podocytes. Podocytes were pretreated with SLx-2119 before stimulation with TGF-β. The scale bar on the top left represents 100 μm (*n* = 3). **d**, Relative mRNA levels of FAO mediators in podocytes treated with SLx-2119 before stimulation with TGF-β (*n* = 3). **e**, PPARα mRNA levels in podocytes treated with SLx-2119 before stimulation with TGF-β. TGF-β mRNA levels in ROCK2 knockdown podocytes (**f**) and in podocytes treated with SLx-2119 (**g**). **h**, Podocytes were treated with TGF-β, and the lysates were subjected to immunoprecipitation with a ROCK2 antibody, followed by Western blotting with a PPARα antibody. Whole cell lysate was analyzed by Western blotting using a ROCK2 antibody (*n* = 3). *$p < 0.05$. Data are represent the mean ± s.e.m.

Fig. 1a

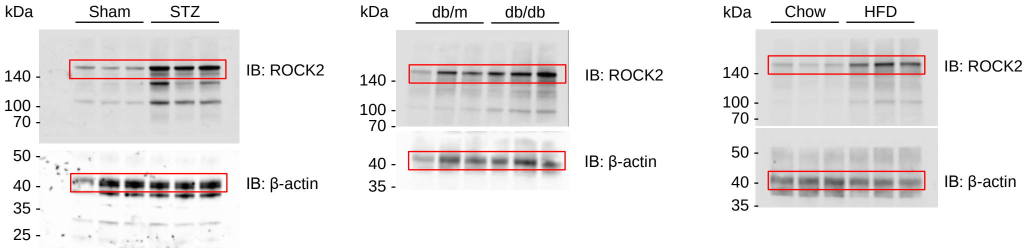

Supplementary Fig. 1b

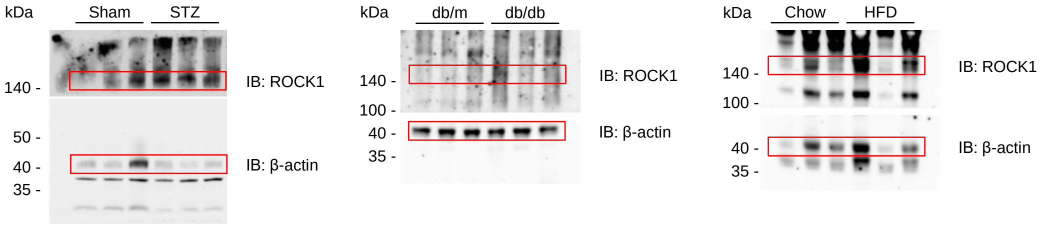

Supplementary Fig. 3b

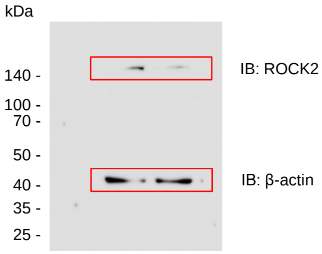

Supplementary Fig. 4h

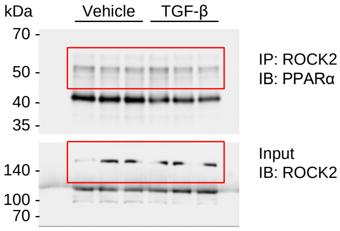

Supplementary Fig. 5: Unedited images. Unprocessed blots for Fig. 1a, Supplementary Fig. 1b, 3b, and 4h.

Tissue type	Age at death (y)	Sex	BMI (kg/m²)	PMI (h)	Cause of death	Comorbidities
Normal #1	52	M	27.7	4.5	Gastric ulcer perforation	Thyroid nodule
Normal #2	50	M	35.4	4	Myocardial infarction	Atherosclerosis
Normal #3	62	M	24.5	3	Heart failure	Chronic alcoholism
Normal #4	68	M	24.5	5	Necrotic paraproctitis	Coronary artery disease
Diabetic nephropathy #1	56	F	27.7	5.5	Ischemic stroke	Atherosclerosis
Diabetic nephropathy #2	90	M	24.8	3	Heart failure	Hypertension
Diabetic nephropathy #3	62	M	26.3	3	Aortic aneurism rupture	Atherosclerosis
Diabetic nephropathy #4	48	M	50.5	4	Gastric ulcer perforation	Metabolic syndrome

Supplementary Table 1: Human kidney sample information. The clinical profiles of the human samples used for the histological examination (Fig. 1e). PMI, Post-mortem interval.

Gene	Organism	GenBank Accession	Forward (5' to 3')	Reverse (5' to 3')
Actb	Mus musculus	NM_007393	GTGACGTTGACATCCGTAAAGA	GCCGGACTCATCGTACTCC
Rock2	Mus musculus	NM_009072	GGTTTACAGATGAAAGCGGAAGA	GTGATGCCTTATGACGAACCAA
Bax	Mus musculus	NM_007527	AGACAGGGGCCTTTTTGCTAC	AATTCGCCGGAGACACTCG
Apaf1	Mus musculus	NM_001042558	CAGTAATGGCGTCTTGTCAGT	AAGCGGCTGCTCGTTGATATT
Cdkn1a	Mus musculus	NM_001111099	CCTGGTGATGTCCGACCTG	CCATGAGCGCATCGCAATC
Tgfb1	Mus musculus	NM_011577	CCACCTGCAAGACCATCGAC	CTGGCGAGCCTTAGTTTGGAC
Fatp1	Mus musculus	NM_017399	ATGAACTTCTCCGGCAAGTACC	GGTCCTCGGGCAGACCTAT
Cpt1a	Mus musculus	NM_013495	TGGCATCATCACTGGTGTGTT	GTCTAGGGTCCGATTGATCTTTG
Cpt1b	Mus musculus	NM_009948.2	GACTTCCGGCTTAGTCGGG	GAATAAGGCGTTTCTTCCAGGA
Cpt2	Mus musculus	NM_009949.2	CAGCACAGCATCGTACCCA	TCCCAATGCCGTTCTCAAAAT
Acox1	Mus musculus	NM_015729	TAACTTCCTCACTCGAAGCCA	AGTTCCATGACCCATCTCTGTC
Acox2	Mus musculus	NM_001161667.1	AACCCAGGGGATCGAGTGT	CGCAGCTCAGTGTTTGGGAT
Acadvl	Mus musculus	NM_017366.3	ACTACTGTGCTTCAGGGACAA	GCAAAGGACTTCGATTCTGCC
Acadl	Mus musculus	NM_007381.4	TTTCCTCGGAGCATGACATTTT	GCCAGCTTTTTCCCAGACCT
Acadm	Mus musculus	NM_007382.5	AACACAACACTCGAAAGCGG	TTCTGCTGTTCCGTCAACTCA
Acads	Mus musculus	NM_007383.3	GACTGGCGACGGTTACACA	GGCAAAGTCACGGCATGTC
Pgc1a	Mus musculus	NM_008904.2	TATGGAGTGACATAGAGTGTGCT	GTCGCTACACCACTTCAATCC
Ppara	Mus musculus	NM_011144	AACATCGAGTGTCGAATATGTGG	CCGAATAGTTCGCCGAAAGAA

Supplementary Table 2: Primer sequences.
