# Supplementary material for: ROCK2-induced metabolic rewiring in diabetic podocytopathy
Source: Commun Biol. 2022 Apr 8;5:341. doi: 10.1038/s42003-022-03300-4 (PMC8993857; doi:10.1038/s42003-022-03300-4)
Supplement: Supplementary file 3 — Description of Additional Supplementary Files [file 42003_2022_3300_MOESM3_ESM.pdf]

Description of Additional Supplementary Files

File name: Supplementary Data 1
Description: Source data underlying the graphs and charts in the main manuscript file.
