# Supplementary material for: ROCK2-induced metabolic rewiring in diabetic podocytopathy
Source: Commun Biol. 2022 Apr 8;5:341. doi: 10.1038/s42003-022-03300-4 (PMC8993857; doi:10.1038/s42003-022-03300-4)
Supplement: Supplementary file 5 — Reporting Summary [file 42003_2022_3300_MOESM5_ESM.pdf]

Corresponding author(s): Keiichiro Matoba

Last updated by author(s): Feb 15, 2022

Reporting Summary

Nature Research wishes to improve the reproducibility of the work that we publish. This form provides structure for consistency and transparency in reporting. For further information on Nature Research policies, see our Editorial Policies and the Editorial Policy Checklist.

Statistics

For all statistical analyses, confirm that the following items are present in the figure legend, table legend, main text, or Methods section.

n/a	Confirmed	
☐	☒	The exact sample size (*n*) for each experimental group/condition, given as a discrete number and unit of measurement
☐	☒	A statement on whether measurements were taken from distinct samples or whether the same sample was measured repeatedly
☐	☒	The statistical test(s) used AND whether they are one- or two-sided<br>*Only common tests should be described solely by name; describe more complex techniques in the Methods section.*
☒	☐	A description of all covariates tested
☐	☒	A description of any assumptions or corrections, such as tests of normality and adjustment for multiple comparisons
☐	☒	A full description of the statistical parameters including central tendency (e.g. means) or other basic estimates (e.g. regression coefficient) AND variation (e.g. standard deviation) or associated estimates of uncertainty (e.g. confidence intervals)
☒	☐	For null hypothesis testing, the test statistic (e.g. *F*, *t*, *r*) with confidence intervals, effect sizes, degrees of freedom and *P* value noted<br>*Give P values as exact values whenever suitable.*
☒	☐	For Bayesian analysis, information on the choice of priors and Markov chain Monte Carlo settings
☒	☐	For hierarchical and complex designs, identification of the appropriate level for tests and full reporting of outcomes
☐	☒	Estimates of effect sizes (e.g. Cohen's *d*, Pearson's *r*), indicating how they were calculated

Our web collection on statistics for biologists contains articles on many of the points above.

Software and code

Policy information about availability of computer code

Data collection	Image acquisition was performed using EVOS M5000 Imaging System (Thermo Fisher Scientific).
Data analysis	ImageJ software was used for western blot quantification. Statistical evaluation was performed using Microsoft Office Excel 2019.

For manuscripts utilizing custom algorithms or software that are central to the research but not yet described in published literature, software must be made available to editors and reviewers. We strongly encourage code deposition in a community repository (e.g. GitHub). See the Nature Research guidelines for submitting code & software for further information.

Data

Policy information about availability of data

All manuscripts must include a data availability statement. This statement should provide the following information, where applicable:
- Accession codes, unique identifiers, or web links for publicly available datasets
- A list of figures that have associated raw data
- A description of any restrictions on data availability

The data that support the findings of this study are available on request from the corresponding author.

Field-specific reporting

Please select the one below that is the best fit for your research. If you are not sure, read the appropriate sections before making your selection.

☒ Life sciences ☐ Behavioural & social sciences ☐ Ecological, evolutionary & environmental sciences

For a reference copy of the document with all sections, see nature.com/documents/nr-reporting-summary-flat.pdf

Life sciences study design

All studies must disclose on these points even when the disclosure is negative.

Sample size	No statistical methods were used to predetermine the sample size. Number of sample was determined based on experimental approach, availability, feasibility required to obtain definitive results.
Data exclusions	No data were excluded from analysis.
Replication	All experiments have been repeated at least three times and/or with sufficient cells/animals/human tissues per group to demonstrate the statistical significance. All data were statistically analyzed.
Randomization	The samples or age-matched male mice were randomly allocated into experimental groups.
Blinding	The investigators were blinded to group allocation during data collection and analysis.

Reporting for specific materials, systems and methods

We require information from authors about some types of materials, experimental systems and methods used in many studies. Here, indicate whether each material, system or method listed is relevant to your study. If you are not sure if a list item applies to your research, read the appropriate section before selecting a response.

Materials & experimental systems

n/a	Involved in the study	
☐	☒	Antibodies
☐	☒	Eukaryotic cell lines
☒	☐	Palaeontology and archaeology
☐	☒	Animals and other organisms
☒	☐	Human research participants
☒	☐	Clinical data
☐	☒	Dual use research of concern

Methods

n/a	Involved in the study	
☒	☐	ChIP-seq
☒	☐	Flow cytometry
☒	☐	MRI-based neuroimaging

Antibodies

Antibodies used	The following antibodies were used for immunostaining: anti-ROCK2 (ab71598), anti-Nephrin (ab227806), and anti-WT1 (ab89901) were purchased from abcam. The primary antibodies used for western blot were as follows: anti-ROCK2 (ab71598) was from abcam; and anti-PPARα (PA1-822A) was obtained from Thermo Fisher Scientific.
Validation	Antibodies were validated by vendors or other researchers.

Eukaryotic cell lines

Policy information about cell lines

Cell line source(s)	E11 cell line was obtained from ATCC.
Authentication	E11 cell lines obtained from ATCC were already authenticated using STR profiling.
Mycoplasma contamination	Mycoplasma testing was not routinely carried out.
Commonly misidentified lines (See ICLAC register)	None.

Animals and other organisms

Policy information about studies involving animals; ARRIVE guidelines recommended for reporting animal research

Laboratory animals	The floxed-ROCK2 mouse line (C57BL/6 background) was generated by inserting the LoxP site flanking exon 3 of the Rock2 gene. Podocyte-specific ROCK2 knockout mice (PR2KO) were generated by crossing ROCK2 flox/flox mice and NPHS2 (also known as Podocin)-cre mice obtained from The Jackson Laboratory. All transgenic mice were genotyped using tail DNA at the timing of weaning. We used ROCK2-floxed, cre-negative littermates as our control. Only male mice were used for studies.
Wild animals	Wild animals were not involved in the present study.
Field-collected samples	Samples were not collected from the field.
Ethics oversight	All experiments involving animals were conducted under protocols approved by the Committee on Ethical Animal Care and Use of The Jikei University School of Medicine.

Note that full information on the approval of the study protocol must also be provided in the manuscript.

Dual use research of concern

Policy information about dual use research of concern

Hazards

Could the accidental, deliberate or reckless misuse of agents or technologies generated in the work, or the application of information presented in the manuscript, pose a threat to:

No	Yes	
☒	☐	Public health
☒	☐	National security
☒	☐	Crops and/or livestock
☒	☐	Ecosystems
☒	☐	Any other significant area

Experiments of concern

Does the work involve any of these experiments of concern:

No	Yes	
☒	☐	Demonstrate how to render a vaccine ineffective
☒	☐	Confer resistance to therapeutically useful antibiotics or antiviral agents
☒	☐	Enhance the virulence of a pathogen or render a nonpathogen virulent
☒	☐	Increase transmissibility of a pathogen
☒	☐	Alter the host range of a pathogen
☒	☐	Enable evasion of diagnostic/detection modalities
☒	☐	Enable the weaponization of a biological agent or toxin
☒	☐	Any other potentially harmful combination of experiments and agents
